# Integrin α6β4 signals through DNA damage response pathway to sensitize breast cancer cells to cisplatin

**DOI:** 10.3389/fonc.2022.1043538

**Published:** 2022-11-10

**Authors:** Min Chen, Brock Marrs, Lei Qi, Teresa Knifley, Heidi L. Weiss, John A. D’Orazio, Kathleen L. O’Connor

**Affiliations:** ^1^ Markey Cancer Center, University of Kentucky, Lexington, KY, United States; ^2^ Department of Toxicology and Cancer Biology, University of Kentucky, Lexington, KY, United States; ^3^ Department of Biostatistics, University of Kentucky, Lexington, KY, United States; ^4^ Department of Pediatrics, University of Kentucky, Lexington, KY, United States; ^5^ Department of Molecular and Cellular Biochemistry, University of Kentucky, Lexington, KY, United States

**Keywords:** integrin signaling, cisplatin sensitivity, triple negative breast cancer, homologous recombination, non-homologous end joining, mutant p53, ATM, DNA-PK

## Abstract

Integrin α6β4 is highly expressed in triple negative breast cancer (TNBC) and drives its most aggressive traits; however, its impact on chemotherapeutic efficacy remains untested. We found that integrin α6β4 signaling promoted sensitivity to cisplatin and carboplatin but not to other chemotherapies tested. Mechanistic investigations revealed that integrin α6β4 stimulated the activation of ATM, p53, and 53BP1, which required the integrin β4 signaling domain. Genetic manipulation of gene expression demonstrated that mutant p53 cooperated with integrin α6β4 for cisplatin sensitivity and was necessary for downstream phosphorylation of 53BP1 and enhanced ATM activation. Additionally, we found that in response to cisplatin-induced DNA double strand break (DSB), integrin α6β4 suppressed the homologous recombination (HR) activity and enhanced non-homologous end joining (NHEJ) repair activity. Finally, we discovered that integrin α6β4 preferentially activated DNA-PK, facilitated DNA-PK-p53 and p53-53BP1 complex formation in response to cisplatin and required DNA-PK to enhance ATM, 53BP1 and p53 activation as well as cisplatin sensitivity. In summary, we discovered a novel function of integrin α6β4 in promoting cisplatin sensitivity in TNBC through DNA damage response pathway.

## Introduction

Cellular context contributes to how tumor cells proliferate, invade, metastasize and respond to therapy. The context of a cell and its microenvironment are read by integrin extracellular matrix receptors, which then integrate these signals for a coordinated response. Epithelial specific integrin α6β4 drives contextual signaling and plays particularly unique roles in tumor progression of various carcinomas, including triple negative breast cancer (TNBC) (1–10).

Integrin α6β4 is a laminin receptor that is more highly expressed in TNBC than in hormone-positive or HER2-amplified breast cancers (11). Furthermore, it is prominently expressed in the basal-like breast cancer subtype (11), which represents about 80% of TNBCs. Integrin α6β4 coordinates and amplifies signals from the microenvironment to drive the most aggressive phenotypes of TNBC by stimulating proliferation, angiogenesis, apoptosis resistance, migration, invasion (10) and metastasis (12, 13). Early investigation on how integrin α6β4 contributes to carcinoma progression linked its signaling to p53, which is notably mutated in 80% of basal/TNBCs (14). In a wildtype p53 background, integrin α6β4 stimulates p53 leading to p21 upregulation, cleavage of Akt and subsequent apoptosis (15–17). In a mutant or null p53 background, however, integrin α6β4 enhances cell survival through stimulation of the PI3K/Akt pathway (15). Our previous work demonstrated that integrin α6β4 signaling epigenetically regulates the expression of pro-invasive genes by stimulating the base excision repair pathway leading to promoter DNA demethylation and can enhance UV-induced nucleotide excision repair (18). These aspects of integrin α6β4 signaling along with other aggressive properties led to the concept that integrin α6β4 would alter therapeutic response (19), but has largely gone unexplored.

Therapies that cause DNA damage remain the standard-of–care for most TNBC patients, although immunotherapy and PARP inhibitors are available for select patients (20). These therapies include ionizing radiation, topoisomerase inhibitors (doxorubicin), alkylating agents (cyclophosphamide), nucleoside analogs (capecitabine/5-fluorouracil (5-FU), gemcitabine) and platinum agents (cisplatin and carboplatin) (21, 22). Chemotherapeutic treatments result in crosstalk between DNA repair pathways that is impacted by cellular context. This phenomenon results in a lack of intuitiveness regarding how to effectively use chemotherapies to target select cancers (23). This concept is exemplified by the requirement of specific chemotherapeutic regimens to treat various types of cancer. As a result, how contextual signaling impacts DNA repair pathway choice and the subsequent patient response to chemotherapies remains an important area of investigation.

In TNBC, homologous recombination (HR) deficiency is a major driver of cisplatin sensitivity, as exemplified by mutations in BRCA1 (24–26). HR and non-homologous end-joining (NHEJ) are the pathways utilized for repairing DNA double strand breaks (DSBs). HR is a high-fidelity repair pathway that requires a DNA template to repair DSBs; accordingly, it is predominantly utilized in S and G2 phases of the cell cycle. NHEJ does not utilize a template and thus can occur in any phase of the cell cycle but is error prone as a result (27). Deciding factors such as 53BP1 define whether HR or NHEJ DNA repair pathways is used for DSB repair. Thus, how these deciding factors are controlled by cellular context can dictate cellular response. In this study, we trace the ability of integrin α6β4 to influence the DNA damage response pathway to promote cisplatin sensitivity in TNBC cells.

## Materials and methods

### Cell lines and drug treatments

All cells were obtained from American Type Culture Collection. BT549 cells were cultured in RPMI1640 (ThermoFisher Scientific, Waltham, ME) containing 10µg/ml insulin (MilliporeSigma, St. Louis, MO). MDA-MB-231 cells were maintained in low-glucose DMEM (ThermoFisher Scientific). MDA-MB-231 cells with inducible knockdown of p53 (28) were cultured in the absence or presence of doxycycline (10µg/ml; MilliporeSigma) to induce p53 silencing. SUM159 cells were cultured in high-glucose DMEM (ThermoFisher Scientific) with 5% FBS, 5µg/ml insulin and 1µg/ml hydrocortisone. All media were supplemented with 10% FBS (MilliporeSigma) if not otherwise specified, 1% L-glutamine, 1% of penicillin and 1% streptomycin (ThermoFisher Scientific). All cell lines, including all derivative cells (ITGB4 and TP53 CRISPR/Cas9 gene editing and retroviral expression) have been authenticated by Laboratory Corporation of America Holdings (LabCorp) using Short tandem repeats (STRs) profiling and confirmed mycoplasma free.

The wildtype full-length integrin β4 construct was obtained from Dr. Livio Trusolino [University of Torino, Italy (29)]. A truncated integrin β4 fragment lacking the signaling domain (β4-1355T) was amplified by PCR using high fidelity Pfu DNA polymerase and cloned into the EcoRI and SalI (New England BioLabs, Ipswich, MA) sites of pBabe-puro vector (Addgene, Cambridge, MA). The primers for β4-1355T are: forward (EcoR1), 5’ CATTAAGAATTCTATGGCAGGGCCACGCCCCA 3’; and reverse (Sal1), 5’ GTA TATGTCGACGCGTAGAACGTCATCGCTGTACATAAG 3’. For stable construct expression, BT549 cells were transfected with empty vector alone or integrin β4 constructs using lipofectamine 2000 and selected with 2 µg/ml of puromycin (ThermoFisher Scientific). The puromycin resistant cells were selected for integrin β4 expression by fluorescence activated cell sorting using the human integrin β4 antibody (clone 439-9B, BD Biosciences, San Jose, CA).

Lentiviral based stable knockdown of β4 integrin expression in SUM149 cells were perform as described previously (30). Dharmacon SMARTPool siRNAs (ThermoFisher Scientific) were electroporated into cells for transient gene suppression as reported previously (31).

Cisplatin, NU7441, and NU7026 were purchased from Selleckchem (Houston, TX). Doxorubicin, gemcitabine, and 5-FU were from MilliporeSigma. Chemotherapeutic agents at the indicated concentrations were added to cells under normal culture conditions. For inhibitor treatment, cells were pretreated with inhibitors for 1h, then cisplatin was added for additional 24h in the presence of inhibitors, as indicated. Since DMSO can affect cisplatin activity, we dissolved cisplatin in normal saline (32).

### CRISPR-Cas9 gene engineering

CRISPR gene engineering utilized pSpCas9(BB)-2A-Puro (PX459) V2.0 (Addgene) and guiding RNA (gRNA) design to knockout ITGB4 and TP53 as described (33). ITGB4 target #454 gRNAs: 5’-CACCGGACATCTGGCTGCGCCGCA-3’ and 5’-AAACTGCGGCGCAGCCAGATGTCC-3’. ITGB4 #661 gRNAs: 5’ CACCGAAATCCAATAGTGTAGTCGC 3’ and 5’AAACGCGACTACACTATTG GATTTC 3’. TP53 gRNAs: 5’ CACCGTCGACGCTAGGATCTGACTG 3’ and 5’ AAACCAGTCA GATCCTAGCGTCGAC 3’. Targeted gRNAs were subcloned into PX459 vector by BBS1 site. Cells were transfected with the gRNAs and CRISPR constructs with TransIT^®^-BrCa Transfection Reagent (Mirus, Madison, WI) and selected with puromycin (0.75 μg/ml). Individual clones were validated by immunoblot analysis for integrin β4 and p53. All CRISPR plasmids were sequenced by Eurofins MWG Operon (Louisville, KY).

### Subcellular protein fractionation and immunoblot analysis

Subcellular fractionation was performed using the Subcellular Protein Fractionation Kit (ThermoFisher Scientific) according to the manufacture’s instruction. Total cell lysates in lysis buffer with phosphatase inhibitors (20mM Tris-HCl (pH 7.5), 150mM NaCl, 1mM Na_2_EDTA, 1mM EGTA, 1% Triton X-100, 2.5mM sodium pyrophosphate, 1 mM β-glycerophosphate, 1mM sodium orthovanadate, 1µg/ml leupeptin, 1mM PMSF) were sonicated, and immunoblotted with various antibodies (Cell Signaling Technology). β-actin (MilliporeSigma) was used as a loading control for total lysates, and tubulin (MilliporeSigma) for cytosolic, p84 (GeneTex, Irvine, CA) for nuclear and histone H2B (Cell Signaling Technology, Beverly, MA) for chromatin bound fractions.

### MTT assay

Cells (1 X 10^3^) were seeded in each well of 96-well plate the day before treatments as noted. After treatment for 6 days, MTT assays were performed in triplicate or greater as reported previously (34) by adding 20 µl MTT (5 mg/ml) to each well and incubated at 37°C for 3 hrs. To dissolve the formazan precipitate, 100 µl of stop solution containing 90% isopropanol and 10% DMSO was added and plates agitated for 20 mins at room temperature and then OD 570 was read. IC50 was calculated using an online tool “Quest Graph™ IC50 Calculator” ATT Bioquest Inc 2021(https://www.aatbio.com/tools/ic50-calculator) and presented as average from at least three independent experiments.

### Clonogenic survival assays

BT549 EV and β4 cells (3 X 10^3^) were seeded into 6-well plate and treated with cisplatin at indicated concentrations for 16 hrs, then cisplatin was rinsed out with PBS. Fresh medium was added, and the cells were allowed to grow for 10 days. Colonies were fixed with methanol for 20 min, stained with 2% crystal violet, and de-stained with water. Colonies with at least 50 cells were counted. Colony survival fractions were calculated as described previously (35). The experiment was performed in triplicate for each treatment condition.

### Immunocytochemistry and the proximity ligation assay

BT549 EV and β4 cells (2.5 X 10^4^) were seeded on glass coverslips coated with 5µg/ml Cultrex mouse laminin-1 (Trevigen, Gaithersburg, MD) overnight and then treated with 10µM cisplatin for 24h. For immunocytochemistry, cells were then fixed, permeabilized, and immunostained as described previously (36) using the following antibodies: p-p53 S15 and p-53BP1 S1778 (Cell Signaling Technology), Cy3- and Cy2-conjugated donkey anti-mouse IgG (Jackson ImmunoResearch, West Grove, PA). DAPI was used to stain nuclei. For PLA assays, cells were fixed and permeabilized according to the Duolink^®^ PLA Fluorescence Protocol (MilliporeSigma). Primary antibodies (1:100, Cell Signaling Technology) used were mouse or rabbit anti-p53, mouse anti-DNA-PKcs, and rabbit anti-53BP1. PLA assays were carried out with Duolink^®^ *In Situ* Detection Reagents Orange (#DUO92007), Duolink^®^ *In Situ* PLA^®^ Probe Anti-Rabbit PLUS/MINUS (#DUO92002/DUO92005) and Duolink^®^ *In Situ* PLA^®^ Probe Anti-Mouse PLUS/MINUS (#DUO92001/DUO92004) from MilliporeSigma. Cells were imaged using a Nikon Eclipse Ti2 Confocal microscope and Nikon NIS Elements software version 3.2.

### DNA repair reporter assays

BT549 cells (EV and β4, 6 X 10^6^) were electroporated (350V, 500µF capacity) with 4µg pDRGFP (HR reporter, Addgene) or 4µg pimEJ5GFP (NHEJ reporter) plus 1.6µg of pmCherry (transfection control) in the presence or absence of 4µg pCBASce-I plasmid (Addgene), which expresses I-SceI endonuclease that creates DSB. Upon repair of the reporter, cells express GFP. After treatment with 5µM cisplatin for 48h, cells (1x10^4^ for each transfection) were analyzed by flow cytometry. The percentage of GFP-positive cells in pmCherry-positive population was used as the indication of DNA repair efficiency for HR or NHEJ.

### Cell cycle analysis by propidium iodide staining

BT549 cells (EV, β4) were plated on laminin-1 coated plates and treated with 10µM cisplatin for 24h. Cells were then trypsinized, rinsed with cold PBS, fixed with cold 70% ethanol, rinsed and then resuspended in PBS staining buffer containing 20µg/ml propidium iodide I, 0.1% Triton X-100, and 200µg/ml RNase A (MilliporeSigma) and incubated at room temperature for 30 min before analyzed the cell cycle distribution by flow cytometry.

### Statistical analysis

Data were compared and analyzed using a two-tailed or one-tailed unpaired Student’s t-test. All experiments were performed at least three times and the representative data were shown and presented as mean ± SD, unless stated otherwise. P values <0.05 between groups were considered significantly different.

## Results

### Integrin α6β4 sensitizes TNBC cells to cisplatin treatment

**Figure 1:**
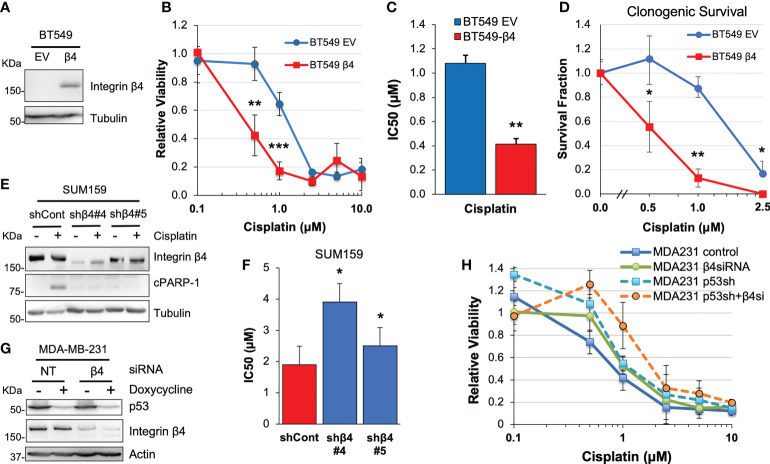
Integrin α6β4 signaling sensitizes cells to cisplatin treatment. BT549 cells expressing EV or wildtype integrin β4 **(A)** were treated with varying doses of cisplatin for 6 days. Cell viability was assessed by MTT assays **(B)** and IC50 was calculated **(C)** or relative clonogenic survival determined **(D)**. **(E, F)** SUM159 cells with integrin β4 shRNA knockdown (shβ4) or control shRNA (shCont) **(E)** were treated with different doses of cisplatin and IC50 calculated from cell viability assays **(F)**. **(G, H)** Mutant p53 was knocked down in a doxycycline-inducible manner in MDA-MB-231 cells and/or integrin β4 was knocked down by siRNA. Cells were then plated on laminin-1-coated plates, treated with various doses of cisplatin for 6 days. Efficiency of target knockdown was monitored by immunoblotting **(G)** and cell viability was assessed by MTT **(H)**. *p < 0.05, **p < 0.001, ***p < 0.0001.

To test how integrin α6β4 signaling influences the response of TNBC cells to chemotherapy, we chose three representative TNBC cell lines with known integrin β4 subunit expression (37–39) and different p53 mutations (40). First, we selected BT549 cells which naturally do not express integrin β4 where we created cells that stably expressed an empty vector (BT549 EV) or the β4 subunit (BT549 β4; **Figure 1A**), which pairs with the endogenous α6 subunit to create the α6β4 integrin (39). We treated the BT549 EV and BT549 β4 cells with various doses of chemotherapeutic agents including cisplatin (**Figures 1B, C**), carboplatin, doxorubicin, gemcitabine, and 5-FU (**Supplemental Figure 1**) for 6 days and then performed MTT assays to assess cell viability. We found there was a three-fold greater sensitivity to cisplatin in cells expressing the integrin β4 (**Figures 1B, C**; 1.1μM IC50 for EV vs 0.4μM for β4). This difference was mirrored in the cells’ response to carboplatin (**Supplemental Figure 1A**), which is reflective of their common mechanism of action (41). A clonogenic survival assay was performed that further confirmed the effect of cisplatin on cell viability (**Figure 1D**). A similar change in IC50 of cisplatin was noted with the shRNA-mediated knockdown of integrin β4 in the SUM159 cells, depending on the efficiency of the shRNA-mediated knockdown (**Figures 1E, F**). Notably, PARP-1 cleavage (cPARP-1) was more prominent in control SUM159 cells that express integrin α6β4 compared to β4 knockdown cells upon cisplatin treatment (**Figure 1D**). However, expression of integrin β4 in BT549 cells had no impact on the response to doxorubicin, gemcitabine, or 5-FU (**Supplemental. Figures 1B-D**). Therefore, we focused the remainder of our study on how integrin α6β4 impacts response to cisplatin in TNBC cells.

Integrin α6β4 is known to signal through p53 in TNBCs where p53 mutation rates are high. To test whether integrin α6β4 cooperated with mutant p53 to alter cisplatin sensitivity, we obtained MDA-MB-231 cells, which have relatively high levels of integrin β4 (42), with doxycycline-inducible knockdown of mutant p53 (28). We induced suppression of p53 in these cells with doxycycline, and/or knocked down integrin β4 expression by siRNA or left cells untreated during cisplatin treatment and then MTT assays were performed. These data revealed that, compared to the knockdown of integrin α6β4 or p53 alone, the effect of knockdown of both mutant p53 and integrin β4 (**Figure 1H**) on cell viability in response to cisplatin is additive (**Figure 1G**; p = 0.03) and highly significant (p < 0.0001 vs control at 1μM and 2.5μM).

### Integrin α6β4 signaling promotes ATM-p53-53BP1 activation and the association of p53 and 53BP1 with chromatin in response to cisplatin treatment

**Figure 2:**
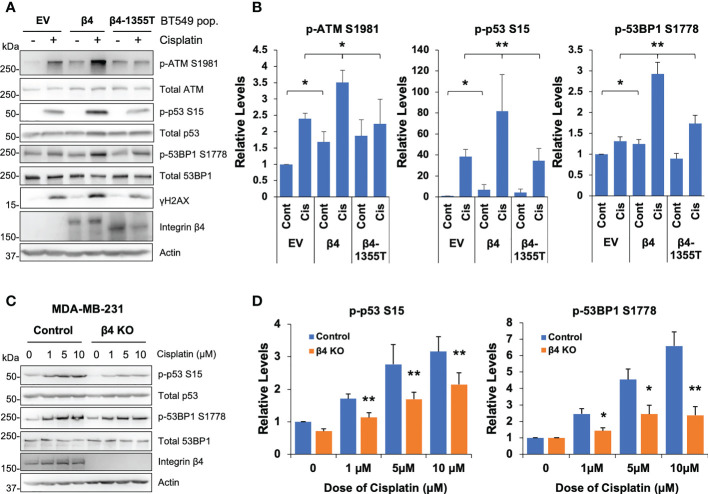
Integrin α6β4 promotes activation of ATM-p53-53BP1 in response to cisplatin that results in enhanced DNA damage. **(A)** BT549 cells (EV, β4, and β4-1355T) were plated on laminin-1 and treated with 10μM cisplatin for 24h prior to assessing for phosphorylation of indicated DDR proteins, as noted. Fold differences in pATM S1981, p53BP1 S1778 and p-p53 S15 were quantified from three separate experiments and reported relative to BT549 EV control **(B)**. **(C)** MDA-MB-231 control or β4 KO (clone 454-1) cells were treated with indicated dose of cisplatin for 24h as in **(A)** and immunoblotted for the indicated phospho-proteins and total proteins. Fold changes in noted phosphorylation sites are reported after normalization to total protein and reported relative to MDA-MB-231 control treatment in **(D)**. *p < 0.05, **p < 0.005.

To determine the impact of integrin α6β4 on cisplatin-mediated DNA damage response (DDR) signaling, we performed cisplatin dose-response and time-course analyses on BT549 EV and β4 cells and investigated the phosphorylation of ATM (S1981), ATR (S1989), p53 (S15), 53BP1 (S1778) and DNA damage marker γH2AX as well as PARP-1 cleavage. The results demonstrated that integrin α6β4 signaling enhanced the amplitude and speed (**Supplemental Figure 2**) of ATM, p53, 53BP1, and H2AX phosphorylation (γH2AX), but not ATR autophosphorylation, in response to cisplatin treatment. In line with its impact on cisplatin sensitivity, integrin α6β4 also enhanced PARP1 cleavage. To test whether the integrin β4 signaling domain was required for the ATM-p53-53BP1 pathway induced by cisplatin, we generated BT549 cells that stably expressed integrin β4 truncation mutation (β4-1355T) in which the signaling domain was deleted (43). Compared to cells expressing wildtype full-length integrin β4, BT549 β4-1355T cells displayed reduced ATM, p53 and 53BP1 phosphorylation that were either similar to or only slightly higher than the BT549 EV cells (**Figures 2A, B**). We also noted that the basal levels of ATM, p53 and 53BP1 phosphorylation are higher in BT549 β4 cells. In contrast, knockout of ITGB4, the gene encoding the β4 subunit, in MDA-MB-231 cells by CRISPR-Cas9 editing resulted in the suppression of p53 and 53BP1 phosphorylation at various doses of cisplatin, thus confirming the role of integrin α6β4 in their activation (**Figures 2C, D**). These observations suggest that integrin α6β4 signaling, specifically through the signaling domain of β4, can enhance the activation of the key proteins in DDR pathway that have the potential to affect how cells respond to cisplatin.

**Figure 3:**
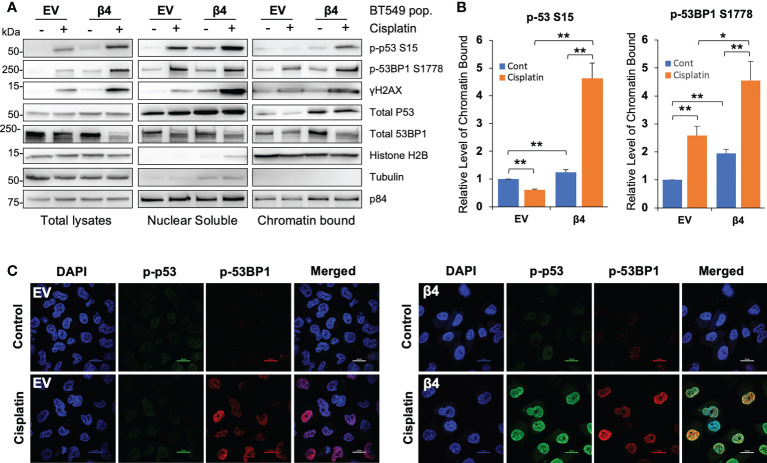
Integrin α6β4 promotes recruitment of p53 and 53BP1 to chromatin. **(A)** BT549 cells (EV and β4) were plated on laminin-1 and treated with 10μM cisplatin for 24h. Subcellular protein fractions were immunoblotted with DDR proteins as indicated. Tubulin was used as the marker for total protein, p84 was used to mark nuclear fractions, and Histone H2B used to mark chromatin fractions. **(B)** Fold changes in noted phosphorylation in chromatin bound from **(A)** are reported after normalization to Histone H2B and reported relative to BT549-EV control treatment. **(C)** Cells treated as in **(A)** were immunostained for phospho-p53 S15 and phospho-53BP1 S1778, as indicated, using DAPI as a counterstain (scale bars, 20µm). *p < 0.05, **p < 0.005.

Next, we sought to define whether the activated p53 and 53BP1 were soluble in the nucleoplasm or associated with chromatin by performing subcellular protein fractionation and immunoblotting for phosphorylated and total p53 and 53BP1. We determined that the associations of p53 S15, 53BP1 S1778 and γH2AX with chromatin were enhanced with integrin β4 expression in response to cisplatin treatment (**Figures 3A, B**). Furthermore, soluble nuclear γH2AX was dramatically increased in BT549 β4 cells upon cisplatin treatment. Interestingly, total p53 levels associated with the chromatin were amplified with integrin α6β4 regardless of the treatment condition. This enhanced chromosomal activation of p53, and that of 53BP1, were also visualized by immunocytochemistry (**Figure 3C**).

Sensitivity to cisplatin and enhanced PARP1 cleavage as a result of integrin α6β4 seemed unexpected based on its role in promoting cell survival and signaling through the Erk and Akt pathways (15, 44), which can contribute to cisplatin resistance (45, 46). Therefore, we investigated the impact of integrin α6β4 signaling on these two survival pathways in conjunction with cisplatin treatment. We found that while the basal activity of Erk was higher in integrin β4 cells compared to EV cells, Erk activation was suppressed upon cisplatin treatment in the BT549 β4 cells but was enhanced in the EV cells. In contrast, the basal phosphorylation of Akt was lower in the BT549 β4 cells and the activation of Akt in response to cisplatin treatment in BT549 EV cells was marginal at low levels of cisplatin but remained unaltered in the BT549 β4 cells (**Supplemental Figure 3**). These data indicate that integrin α6β4 signaling to survival pathways is attenuated during cisplatin treatment.

### Mutant p53 facilitates integrin α6β4-mediated ATM/53BP1 activation and sensitization to cisplatin

**Figure 4:**
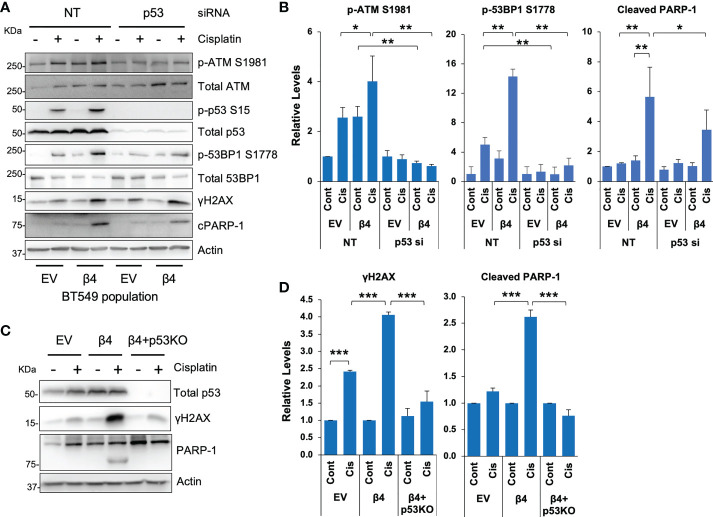
Mutant p53 is required for cisplatin-induced ATM and 53BP1 activations and integrin α6β4-mediated sensitivity to cisplatin. P53 was knocked down by siRNA in BT549 EV and BT549 β4 cells **(A, B)** or knocked out by CRISPR/Cas9 in BT549 β4 cells **(C, D)**, then cells were plated on laminin-1-coated plates and treated with 10µM cisplatin for 24h prior to harvesting for immunoblotting analysis **(A, C)**. Fold differences were quantified from three separate experiments, normalized to respective total protein (for p-ATM S1981 and p-53BP1 S1778) or actin for cleavage PARP-1 (cPARP-1) and γH2AX and reported relative to their respective untreated controls indicated **(B, D)**. *p < 0.05, **p < 0.005, ***p < 0.001.

Most TNBCs (14) have mutant p53 with gain-of-function properties (47), including BT549 and MDA-MB-231 cell lines. To test the requirement for mutant p53 in cisplatin-induced DDR, we knocked down p53 by siRNA in BT549 EV and integrin β4 cells, treated these cells with cisplatin and then assessed DNA repair pathways. We show that knockdown of p53 blocked activation of ATM in response to cisplatin and/or integrin α6β4 signaling as well as the downstream 53BP1 phosphorylation (**Figures 4A, B**). We also noted that compared to the non-targeting control (NT), knockdown of p53 in BT549-β4 cells had less PARP-1 cleavage with cisplatin treatment, while knockdown of p53 in BT549-EV cells did not affect the PARP-1 cleavage in response to cisplatin (**Figures 4A, B**). The impact of mutant p53 downstream of integrin α6β4 was confirmed with CRISPR-Cas9 knockout of p53 in the BT549-β4 cells where p53 KO blocked the enhanced γH2AX and PARP-1 cleavage mediated by integrin α6β4 (**Figures 4C, D**). Collectively, these results demonstrate that mutant p53 is needed for the amplification of ATM activity and 53BP1 phosphorylation downstream of integrin α6β4 signaling in response to cisplatin and is an integral component of integrin α6β4-enhanced cisplatin sensitivity.

### Integrin α6β4 signaling stimulates NHEJ and suppresses HR in response to cisplatin

**Figure 5:**
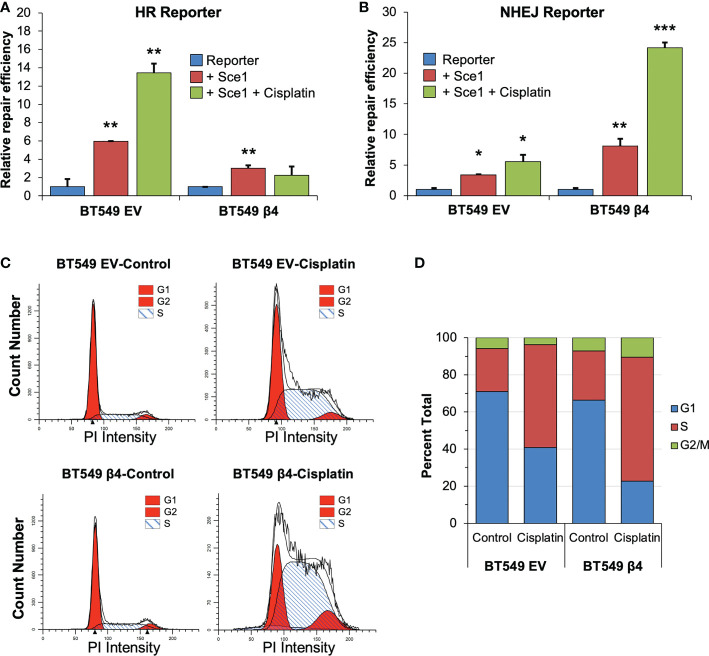
Integrin α6β4 signaling enhances NHEJ and suppresses HR. BT549 cells (EV, β4) were electroporated with pDRGFP (HR reporter; A) or pimEJ5GFP (NHEJ reporter; B) in the presence or absence of pCBASce-I plasmid, which expresses I-SceI endonuclease that causes DSB, plus pmCherry as a transfection control. After cells were plated on laminin-1-coated plates and treated with 5µM cisplatin for 48h, cells were analyzed GFP positive cells by flow cytometry using cotransfected pmCherry as a transfection control to determine relative repair efficiency **(A, B)**. **(C, D)** BT549 cells (EV, β4) plated on laminin-1 coated plates were treated with 10µM cisplatin for 24h and assessed for cell cycle distribution using propidium iodide staining and flow cytometry analysis. Representative **(C)** and averaged **(D)** cell cycle distributions are shown. Data are representative of 3 experiments. *p < 0.05, **p < 0.001, ***p < 0.0001.

53BP1 is important for the DNA repair choice between HR and NHEJ that influences cisplatin response (48). To test if integrin α6β4 signaling could alter DNA repair pathway choice, we utilized commonly used HR and NHEJ reporter systems that use the endonuclease Sce-1 to cause DSBs that, upon repair, create a functional GFP molecule. Here, BT549 EV and integrin β4 cells were co-transfected with pDRGFP (HR reporter (49) or pimEJ5GFP (NHEJ reporter (50)) in the presence or absence of pCBASce-I and with pmCherry as an internal transfection control. Transfected cells were then treated with or without 5μM cisplatin for 48h and analyzed for GFP and mCherry by flow cytometry. As shown in **Figures 5A, B**, BT549 EV cells had a higher basal HR activity than the integrin β4 cells. Conversely, the integrin β4 cells displayed higher basal level of NHEJ activity than the EV cells. Cisplatin treatment dramatically activated HR activity in BT549 EV cells but suppressed HR in integrin β4 expressing cells. In contrast, NHEJ activity in BT549 β4 cells was dramatically activated upon cisplatin treatment compared to the EV cells.

NHEJ is known to function as the primary DSB repair mechanism in G1, while both NHEJ and HR occur in S and G2 phases (27). To test whether integrin α6β4 signaling shifts DNA repair pathway from HR to NHEJ by impacting cell cycle distribution during cisplatin treatment (specifically G1 arrest), we investigated the cell cycle distribution of cells under these conditions. In untreated cells, we found that the cell cycle distribution was similar between the EV and β4 expressing cells, except BT549 β4 cells had slightly more cells in S and G2/M phases. When cells were treated with cisplatin, both cell populations showed a 2.5-fold increase in S phase and a concomitant drop in G1 distribution, which was more pronounced in the BT549 β4 cells. In contrast, BT549 EV cells lost approximately 40% of their G2 distribution, while the β4 cells doubled their G2 distribution (**Figures 5C, D**). These data indicate that cisplatin treatment causes an accumulation of cells in S-phase of the cell cycle and a loss of cells in G1 that is indicative of replication stress instead of a G1 arrest.

### DNA-PK is preferentially activated downstream of integrin α6β4 and required for enhanced 53BP1 phosphorylation

**Figure 6:**
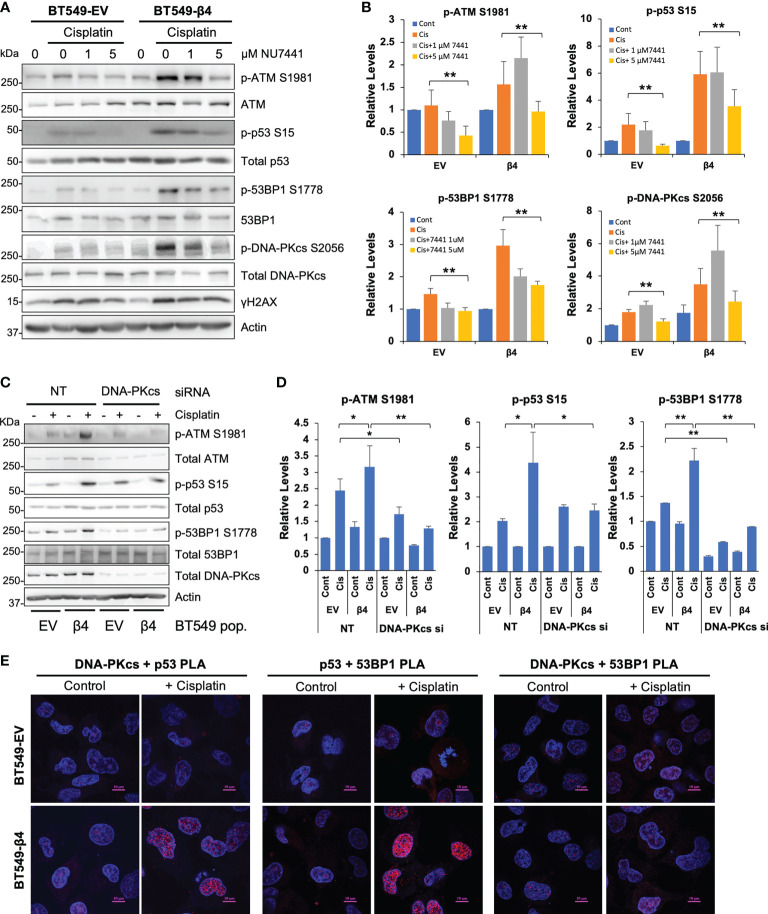
DNA-PK is activated and required for phosphorylation of ATM, 53BP1 and p53 downstream of integrin α6β4. BT549 cells (EV and β4) were plated on laminin-1-coated plates, pretreated with DNA-PK inhibitors NU7441 **(A)** at indicated concentrations for 1 hr, or electroporated with 200nM non-targeting siRNA or siRNA targeting DNA-PKcs for 72h **(C)** before treatment with 10µM cisplatin for 24h. Cell lysates were then immunoblotted for signaling proteins in DNA repair pathway as noted and quantified as fold change compared to total protein controls **(B, D)**. *p < 0.05, **p < 0.005. **(E)** BT549 cells (EV and β4) plated on laminin-1-coated coverslips were treated with 10µM cisplatin for 24h and then the associations of DNA-PKcs, p53, and 53BP1 were assessed by PLA, as noted. Scale bars, 10µm.

p53 and 53BP1 (51) are targets of DNA-PK, a DNA damage sensing kinase involved in NHEJ DSB repair. Accordingly, we assessed how cisplatin and integrin α6β4 signaling impact DNA-PK activation and the influence of DNA-PK inhibition on downstream DDR signaling. As shown in **Figure 6** and **Supplemental Figure 4**, cisplatin treatment resulted in DNA-PKcs phosphorylation at S2056 and T2609, which are indicative of an activated kinase, that was substantially greater in BT549 β4 cells than in EV cells. To determine how DNA-PK activity affects cisplatin-induced DNA repair pathways, we pretreated BT549 EV and integrin β4 cells with DNA-PK inhibitors NU7441 (**Figures 6A, B**) or NU7026 (**Supplemental Figure 4**) at various concentrations prior to cisplatin treatment. We found that phosphorylation of 53BP1 in response to cisplatin treatment was particularly sensitive to DNA-PK inhibition, suggesting that DNA-PK controls 53BP1 phosphorylation. We found that knockdown of DNA-PKcs by siRNA substantially decreased the activation of ATM, 53BP1 and p53 in response to cisplatin (**Figures 6C, D** **),** consistent with the results from DNA-PK inhibitors. To investigate whether DNA-PK forms complex with 53BP1 and p53 in response to cisplatin and how integrin α6β4 influences these associations, we performed PLAs for DNA-PKcs-p53, p53-53BP1, and DNA-PKcs-53BP1 complexes with and without cisplatin treatment. We found that DNA-PKcs-p53 complexes and p53-53BP1 complexes formed preferentially in the integrin β4 expressing cells after cisplatin treatment; however, DNA-PKcs did not appear to complex directly with 53BP1 (**Figure 6E**). These data, coupled with our observation that mutant p53 was required for 53BP1 activation (**Figure 4**), suggest that integrin α6β4 signaling to DNA-PK controls 53BP1 phosphorylation in response to cisplatin by activating and recruiting p53 to link DNA-PK to 53BP1.

**Figure 7:**
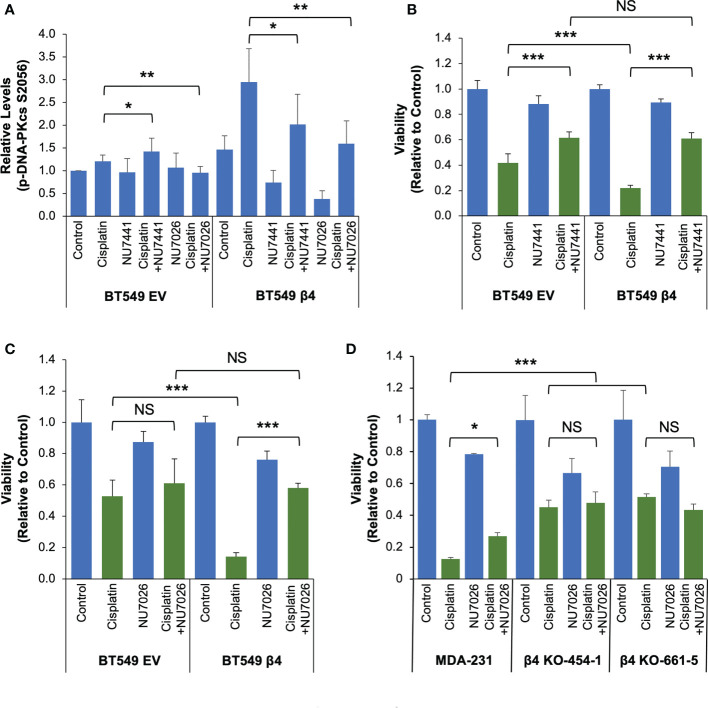
DNA-PK is critical for integrin α6β4 signaling-mediated cisplatin sensitivity. **(A-C)** BT549 cells (EV and β4) were treated with vehicle (Control), 1µM cisplatin and/or DNA-PK inhibitor (0.1 µM NU7441 or 1µM NU7026, as noted) for 6 days. The phosphorylation of DNA-PKcs S2056 was assessed by immunoblotting, quantified, and reported as relative changes to BT549-EV control **(A)**. **(D)** MDA-MB-231 cells and the integrin β4 knock out cells with one of two distinct gRNAs (clones 454-1 and 661-5) were treated with vehicle (Control), 1µM cisplatin and/or 1µM NU7026 as noted for 6 days. Cell viability was assessed by MTT assays for the effect of NU7441 **(B)** and NU7026 **(C, D)** for indicated cells. Data are representative of at least 3 separate experiments. *p < 0.05, **p < 0.005. ***p < 0.0005. NS, Not Significant.

In order to investigate the impact of DNA-PK activation on integrin α6β4 signaling-mediated cisplatin sensitivity, we first performed the dose-dependent studies on cell viability using two DNA-PK inhibitors NU7441 and NU7026 (**Supplemental Figure 5** **)** with different potency. We selected the concentrations (NU7441, 0.1µM; or NU7026, 1µM) that resulted in only a modest reduction in cell viability over a 6-day period (**Supplemental Figure 5**) for use in combination treatments with cisplatin. At these concentrations, we noted that both DNA-PK inhibitors reduced DNA-PKcs phosphorylation in BT549-β4 cells; however, the response in BT549-EV cells was modest and variable (**Figure 7A** and **Supplemental Figure 6**). We treated BT549 EV and β4 cells with vehicle (control), 1µM cisplatin only, cisplatin plus DNA-PK inhibitor, or DNA-PK inhibitor alone for 6 days and cell viability was assessed. As quantified in **Figures 7B, C**, compared to cisplatin treatment alone, adding DNA-PK inhibitor increased cell viability, although the effect in EV cells was either modest (NU7441) or not significant (NU7026). In contrast, in BT549 β4 cells treated with cisplatin, DNA-PK inhibition erased the sensitivity afforded by integrin α6β4 signaling. Notably, the cisplatin response for the EV cells with and without DNA-PK inhibitor and the β4 cells with inhibitor displayed similar viability (**Figures 7B, C**). Notably, the same experiment performed with MDA-MB-231 cells and two β4 KO clones yielded comparable results (**Figure 7D**). These data suggest that the ability of integrin α6β4 to enhance DNA-PK activation in response to cisplatin is responsible for the cisplatin sensitivity observed in these cells.

## Discussion

Despite the observation that select chemotherapies cause similar types of DNA damage, individual cancers from various organ sites do not respond to these chemotherapies in the same way. This observation suggests that the DNA damage response extends beyond the assembly of repair machinery in the nucleus and is impacted by how a cell responds on the cellular level (23). The tumor microenvironment is known to impact DNA repair through factors such as extracellular matrix (52), chronic hypoxia, inflammation and immune regulation (53). These factors influence genomic stability, apoptosis, and DNA repair pathway choices that affect therapeutic efficacy. Integrins as receptors for the extracellular matrix allow cells to sense their environment, yet little is known regarding how they impact the DNA repair process.

The concept that integrins can affect the functions within the nucleus has been long established. Mechanistically, integrins and their connections to the nucleus have been shown to alter nuclear morphology resulting from mechanotransduction (54, 55) and are required for processes such as cyclin D accumulation required to pass the cell cycle checkpoints so cells can enter into mitosis (56, 57). We (30, 58) and others (59, 60) have also shown that integrins, and integrin α6β4 specifically, can impact transcription factors and epigenetics to influence the transcriptome. Our work has shown that integrin α6β4 can influence DNA methylation mediated through base excision repair (58). While integrin α6β4 can bind multiple cytoskeletons that associate with various Nesprins that link the nucleus mechanically, the linkages used by integrin α6β4 specifically can connect to the nucleus are not yet mapped. Our study provides therapeutically important readouts that will facilitate the dissection of these structures important for this signaling required for future studies.

Our study implicates integrin α6β4 in the DNA damage response. Specifically, we provide evidence that integrin α6β4 signaling can integrate cisplatin-induced DNA damage response pathway involving activation of the DNA-PK - mutant p53-53BP1 pathways and alteration of the DSB pathway choice. We also demonstrated that both mutant p53 and DNA-PK activation are critical for integrin α6β4 signaling to promote cisplatin sensitivity in TNBC cells. A recent study in colorectal cancer demonstrated that integrin α6β4 stimulates p53 phosphorylation in response to cisplatin and that integrin β4 knockdown caused resistance to platinum treatment in cells in which wildtype p53 is stabilized (61). This finding is in line with previous work suggesting integrin α6β4 signaling through wild-type p53 promotes apoptosis (16). In TNBCs, however, mutant p53 and integrin α6β4 signaling in cisplatin response may function preferentially in the DNA damage response.

Traditional thought recognizes that p53 promotes DNA repair by promoting cell cycle arrest, thus giving cells time to recover, or promoting apoptosis if repair cannot be achieved. These properties of wildtype p53 depend on p53’s transcriptional activities, which are predominantly lost with most p53 mutations. However, p53 also participates in DNA repair, including NHEJ and HR, that can be independent of DNA binding and transcriptional activity (62). Interestingly, p53 can restrict HR during S phase replication stress (63) through DNA-PK-mediated phosphorylation and subsequent disruptions of p53 and its interactions with RPA (64). These observations suggest that p53 can influence the crosstalk between the HR and NHEJ pathways even when p53 is mutated. In fact, it was found that the interaction of the p53 mutants with MRE11 in the MRN complex prevent the association of the MRN complex to the DSB, resulting in replication stress and impaired DNA-damage response (65, 66). We have found that mutant p53 is highly active in the nucleus in response to cisplatin in integrin α6β4 expressing TNBC cells; however, the levels of total p53 associated with chromatin were amplified with integrin α6β4 regardless of the treatment condition (**Figure 3**). Cisplatin causes replication stress due to interstrand DNA crosslinking (67). Our data strongly support that the mutant p53 and DNA-PK are critical to integrin α6β4 signaling-mediated cisplatin sensitivity in TNBC cells. We also demonstrated that integrin α6β4 signaling results in accumulation of cells in S phase with cisplatin treatment (**Figure 6**) that is indicative of replication stress, thus suggesting how integrin α6β4 signaling through DNA-PK and p53 might be able to suppress HR and increase sensitivity to cisplatin. However, it is unclear whether integrin α6β4 signaling through mutant p53 directly signals to enhance the activation of ATM and DNA-PK, causes enhanced damage due to disruption of cell cycle checkpoints or a combination of these two mechanisms.

A study by Heijink et al. used systems-level methodology that combined quantitative time-resolved signaling data, phenotypic responses, and mathematical modeling to determine key mediators of cisplatin sensitivity. They found that defects in DNA damage response were not required for cisplatin sensitivity but rather signaling dynamics and inactivation of cell cycle checkpoint regulation were the determinants of cisplatin sensitivity (68). Our data are in line with the observation that cells do not need to be defective in DDR to provide cisplatin sensitivity. It is also possible that a shift from HR to NHEJ, and loss of cell cycle checkpoints cooperate and ultimately lead to the accumulation of DNA damage that Telli et al. (69) suggested is prognostic for cisplatin sensitivity. Whether integrin α6β4 signaling alters only these cisplatin-induced signaling dynamics that amplify DNA-PK activity or also contributes to cell cycle checkpoint deficiency will require further investigation.

HR-mediated DNA repair deficiency, typified by BRCA1 mutations, has the strongest association with efficacy of platinum-based therapies in TNBC and is a major determinant of which patients receive platinum regimens (70, 71). Despite its ability to shuttle DSB repair from HR to NHEJ, DNA-PK is not conceptually associated with HR-deficiency. Our data demonstrate that integrin α6β4 signaling through DNA-PK promotes 53BP1 phosphorylation and contributes to cisplatin sensitivity. Notably, we find that inhibition of DNA-PK with two different chemical inhibitors reverses the sensitivity attributed to integrin α6β4, thus suggesting DNA-PK is a mediator of cisplatin sensitivity downstream of integrin α6β4 in TNBC cells. 53BP1 is a major down-stream mediator of p53 and DNA-PK that has been implicated in the decision between HR and NHEJ that can alter sensitivity to cisplatin (48, 72, 73). Notably, loss of 53BP1 has been attributed to cisplatin resistance in BRCA1 mutant cells (74). While it has been unclear how mutant p53 impacts 53BP1 function (72), our data suggest that mutant p53 downstream of integrin α6β4 signaling brings 53BP1 in close proximity with DNA-PK to allow it to be phosphorylated on multiple sites to potentiate its function in stimulating NHEJ. Mutant p53, which is found in 80% of TNBCs (14), has been documented to promote either sensitivity or resistance to cisplatin depending on biological context (47, 75, 76), including direct blockade of the HR pathway (66). Our data indicate that integrin α6β4 may provide that context and promote cisplatin sensitivity by forcing DSB repair to the NHEJ pathway through the mutant p53-53BP1 interactions. However, it is important to note that platinum can cause interstrand crosslinks that can result in DSBs as well as intrastrand crosslinks that create platinum adducts that are repaired by the NER pathway through ATR. While we find that ATR autophosphorylation upon cisplatin treatment is not significantly elevated in response to integrin α6β4 signaling, we cannot conclude conclusively that ATR does not play a role in the platinum response downstream of integrin α6β4 and may require further investigation.

**Figure 8:**
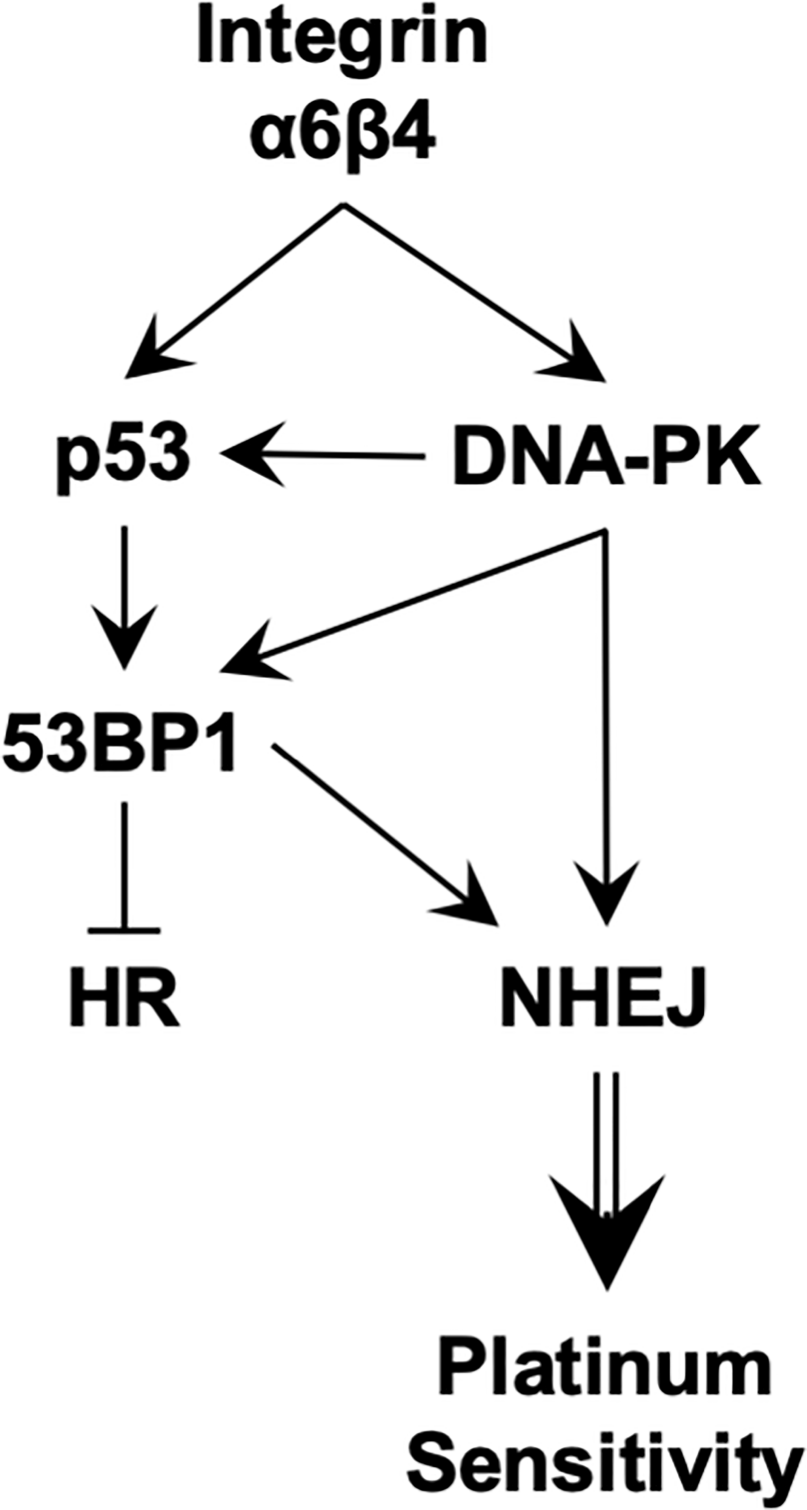
Proposed signaling downstream of integrin α6β4 that promotes sensitivity to platinum agents.

In summary, we trace the ability of integrin α6β4 to affect cisplatin sensitivity to its signaling through mutant p53, amplifying ATM and DNA-PK activity, increasing 53BP1 phosphorylation, and preferentially activating NHEJ over HR in DSB repair (**Figure 8**). This signaling through DNA-PK and activation of the NHEJ pathway appears to lead to suppression of HR that characterizes TNBC and their response to select chemotherapies. Together, this study places the integrin α6β4 signaling cascade as an important regulator of cisplatin sensitivity in TNBC. Future studies to decipher how integrin α6β4 signals to mutant p53, impacts the DNA-PK-p53-53BP1 pathway, and influences DSB repair pathway choice will be vital for understanding the biology of TNBC response to cisplatin.

## Data availability statement

The raw data supporting the conclusions of this article will be made available by the authors, without undue reservation.

## Author contributions

All authors listed have made a substantial, direct, and intellectual contribution to the work and approved it for publication.

## Funding

This work was supported by the National Institutes of Health through National Cancer Institute (R01 CA223164-01 to KO’C, R21CA178753 to KO’C, and R01CA131075 to JD’O); the University of Kentucky Center for Cancer and Metabolism (P20GM121327) for providing imaging services and pilot funding (no number, to MC); by a Markey Women Strong Award through the Markey Cancer Foundation (no number, KO’C); and by the University of Kentucky Markey Cancer Center’s Support Grant (P30CA177558) to provide pilot funding (no number, to MC) and to enable services from the Markey Cancer Center Biospecimen Procurement and Translational Pathology, Biostatistics and Bioinformatics, and Flow Cytometry and Immune Monitoring Shared Resource Facilities.

## Acknowledgments

We gratefully acknowledge Drs. Eva Goellner, Tadahide Izumi, and David Orren for thoughtful discussions. We thank Drs. Jill Bargonetti and Livio Trusolino for reagents.

## Conflict of interest

The authors declare that the research was conducted in the absence of any commercial or financial relationships that could be construed as a potential conflict of interest.

## Publisher’s note

All claims expressed in this article are solely those of the authors and do not necessarily represent those of their affiliated organizations, or those of the publisher, the editors and the reviewers. Any product that may be evaluated in this article, or claim that may be made by its manufacturer, is not guaranteed or endorsed by the publisher.
