# Supplementary material for: Integrin α6β4 signals through DNA damage response pathway to sensitize breast cancer cells to cisplatin
Source: Front Oncol. 2022 Nov 10;12:1043538. doi: 10.3389/fonc.2022.1043538 (PMC9686853; doi:10.3389/fonc.2022.1043538)
Supplement: Supplementary file 1 [file Presentation_1.pdf]

Supplemental Figures

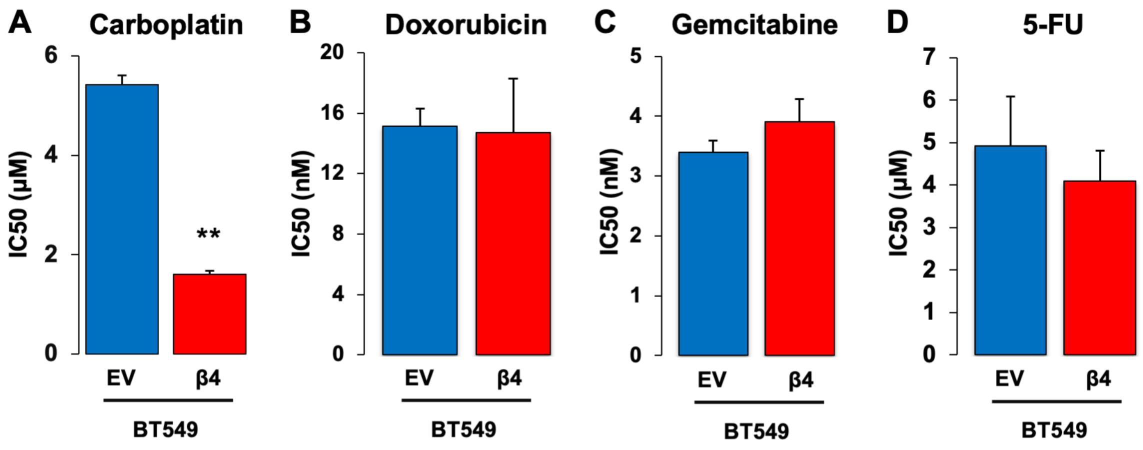

Supplemental Figure 1. Integrin α6β4 signaling sensitizes cells to carboplatin but not to the other chemotherapeutic agents tested. BT549 cells expressing EV and wildtype β4 were treated with varying doses of chemotherapeutic agents as indicated for 6 days. Cell viability was assessed by MTT assays and IC50 was calculated for carboplatin (A), doxorubicin (B), gemcitabine (C), and 5FU (D). ** $p<0.001$ when comparing BT549 EV vs β4 cells; no statistical differences were noted in B-C.

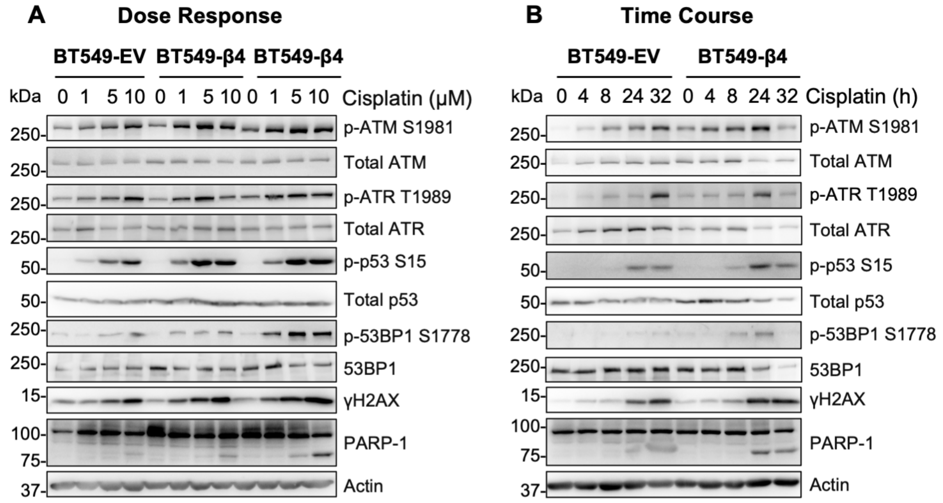

Supplemental Figure 2. Integrin α6β4 promotes activation of ATM-p53-53BP1 pathway in a dose and time dependent manner in response to cisplatin that results in enhanced DNA damage and PARP1 cleavage. (A, B) BT549 cells (EV and β4) were plated on laminin-1, treated with the indicated dose of cisplatin for 24h (A) or 10µM cisplatin for indicated times (B) and assessed proteins or phosphoproteins as indicated.

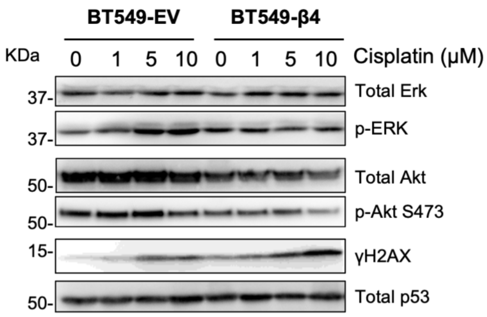

Supplemental Figure 3. Impact of Integrin α6β4 on Akt and Erk signaling in response to cisplatin. BT549 cells (EV and β4) were plated on laminin-1, treated with the indicated dose of cisplatin for 24h and then cell lysates were assessed for phospho-Erk, phospho-Akt, γH2AX, and total Erk, Akt and p53.

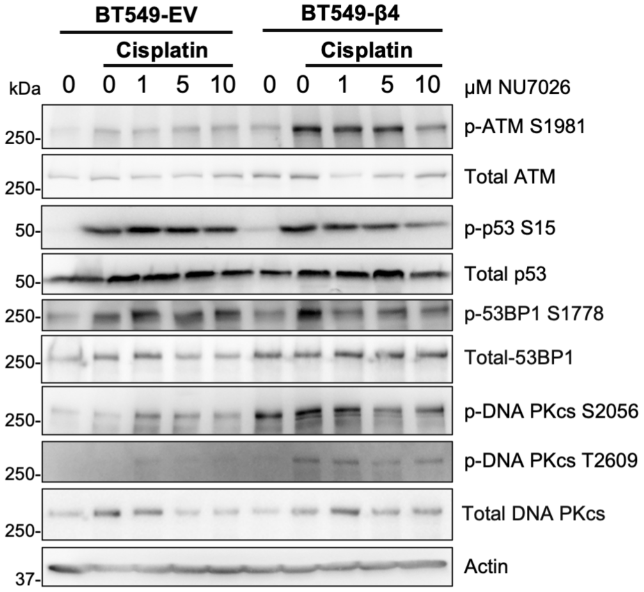

Supplemental Figure 4. Impact of DNA-PK inhibitor, NU7026 on ATM, p53, 53BP1 and DNA-PK downstream of integrin α6β4 signaling. BT549 cells (EV and β4) were plated on laminin-1-coated plates, pretreated with DMSO or DNA-PK inhibitor NU7026 at indicated concentrations for 1 hr before treatment with 10μM cisplatin. Cell lysates were then immunoblotted with signaling proteins in DNA-repair pathway as noted.

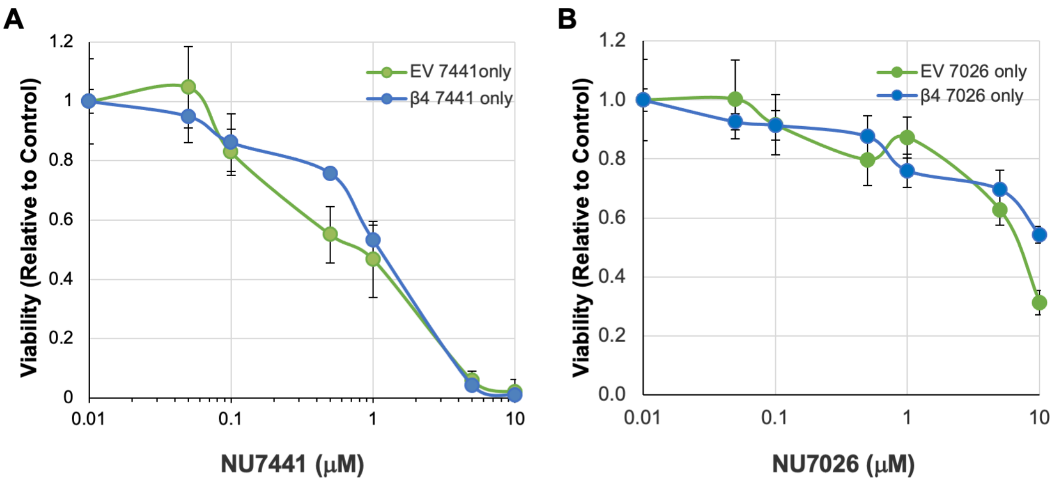

Supplemental Figure 5. Dose response curves of DNA-PK inhibitors, NU7441 and NU7026. Cells, as noted, were treated with DMSO (control) or DNA-PK inhibitors, NU7441 or NU7026 at the indicated dosages for 6 days and assessed for viability by MTT assays. Viability is reported relative to DMSO treated controls.

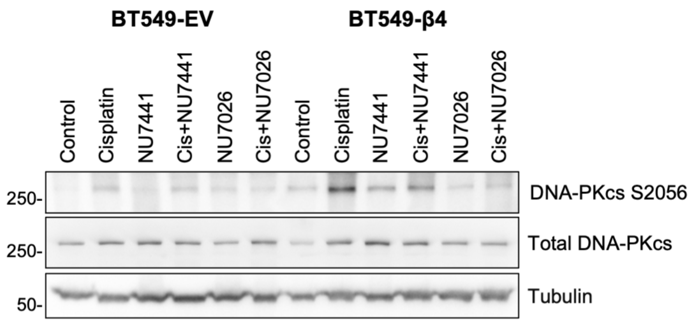

Supplemental Figure 6. Effect of DNA-PK inhibitors on DNA-PKcs phosphorylation. Cells as indicated were treated with vehicle (Control), 1μM cisplatin and/or DNA-PK inhibitor (0.1 μM NU7441 or 1μM NU7026, as noted) for 6 days. Cell lysates then were assessed for phosphorylation of DNA-PKcs S2056, total DNA-PKcs, and tubulin.
